# Oncologic patients’ misconceptions may impede enrollment into clinical trials: a cross-sectional study

**DOI:** 10.1186/s12874-021-01478-5

**Published:** 2022-01-07

**Authors:** Nethanel Asher, Ari Raphael, Ido Wolf, Sharon Pelles, Ravit Geva

**Affiliations:** 1 The Ella Lemelbaum Institute for Immuno-Oncology, Sheba Medical Center, Tel Hashomer, Israel; 2 Sackler Faculty of Medicine Tel Aviv University Tel Aviv, Tel Aviv, Israel; 3 The Oncology Division Clinical Trials Unit, Tel Aviv Sourasky Medical Center, 6 Weizmann St, 6423906 Tel Aviv, Israel

**Keywords:** Cancer clinical trial, Accrual strategies, Patient knowledge, Cancer patient misconception, Cancer patient education

## Abstract

**Background:**

Clinical trials are an essential source for advances in oncologic care, yet the enrollment rate is only 2-4%. Patients' reluctance to participate is an important barrier. This study evaluates patients' level of understanding and attitudes towards clinical trials.

**Methods:**

This cross-sectional study was conducted in the oncology department and day care unit at the oncology division Tel Aviv Sourasky Medical Center, Israel. From January 2015 to September 2016. Two-hundred patients’ currently receiving active anti-cancer therapy at a large tertiary hospital completed an anonymous questionnaire comprised of demographic information, past experience in clinical research and basic knowledge on clinical trials.

**Results:**

The majority of respondents did not meet the minimum knowledge level criteria. In those who replied they would decline to participate in a clinical trial, concern were related to potential assignment to the placebo arm, provision of informed consent and trust issues with their oncologist. Those with sufficient knowledge were significantly more interested in participating. Patients with past experience in clinical trials had a higher level of academic education, were less religious, had a better understanding of medical research and were inclined to participate in future research.

**Conclusions:**

Misperceptions of clinical trials may contribute substantially to the unwillingness to participate in them.

## Background

Clinical trials are the cornerstone of advances in clinical oncology and essential for the evaluation of novel therapies and treatment strategies. The success of clinical trials depends upon adequate patient recruitment, but it has been reported as being as low as 2-4% of all oncologic patients [1]. Unger and colleagues analyzed data from 1,262 cancer patients and found that only 12%-17% of eligible patients eventually participated in clinical studies [2]. It is estimated that about 60% of the currently >10,000 recruiting oncologic clinical trials would enroll less than 5 participants at each site, and more than 20% would enroll none. As a result, only one in 5 recruiting clinical trials would eventually be achievable [3].

This extremely low rate of recruitment may be attributed to several factors. The major reasons are the limited availability of appropriate trials and the highly specific eligibility requirements [4], together accounting for the preclusion of up to 75% of the oncologic population from participation [5]. Other barriers are attributable to the reservations of the treating oncologist, such as ethical dilemmas, insecurity regarding patient recruitment for early phase research and ageism [6–8]. Finally, as many as 15% of the oncologic population refuses outright to participate [5].

Patient refusal may be due to practical considerations, such as time commitment or transportation requirements. Certain socioeconomic and demographic characteristics are also linked with the likelihood of consent [9–11]. Specifically, patients with lower income, low education level, as well as the senior population (over 75 years) are less likely to agree to participate in a clinical trial [12, 13]. Cultural differences are also reported as an important barrier to participation, mainly due to distrust of the medical community [13]. Other significant barrier is patients’ poor understanding of the rationale behind clinical trials [14]. Key areas of conflict involve concerns regarding the informed consent process [15–17], the unease with the randomization process and the fear of being assigned to the placebo arm [4], the sense of losing control and of being "a guinea pig" [18], concerns regarding potential adverse events and their possible impact on quality of life [12], and interestingly, trust issues with the treating oncologist [19].

The limited understanding of the clinical trial process itself, however, is the most definitive reason for noncompliance and it is mainly due to lack of knowledge and sparse information given by the caring physician, according to Center for Information and Study on Clinical Research Participation (CISCRP) [20]. Many studies suggested that the lack of available information that is presented in a clear manner to the potential participants as well as to their treating physicians poses a significant barrier to the success of a recruitment process [21–24].

Educational patient- and physician-centered programs were demonstrated to improve the knowledgeability of the physicians and their readiness to share the information with their patients as well as the patients' willingness to participate in clinical trials. However, the relative importance of the specific issues addressed in the educational programs and their impact on the final decision to participate in a clinical trial have not been studied in depth.

Our hypothesis is that patients’ lack of knowledge and misconception about clinical trials is the main reason why they do not participate in them.

The aim of our study, therefore, was to identify the key issues which significantly contribute to the refusal to participate in clinical trials. We evaluated the level of knowledge about clinical trials and the attitudes towards them on the part of the oncologic population at the Tel Aviv Sourasky Medical Center (TASMC), Israel. The TASMC is a tertiary center with over 4,000 new oncologic patients per year and an adjacent clinical research unit running phase I to phase IV clinical trials. We sought to answer the question of whether selected misconceptions of clinical trials among patients currently receiving anti-cancer treatment pose more significant barriers to clinical trial recruitment.

## Methods

In this cross-sectional study, we conducted a survey among patients diagnosed with cancer who were actively undergoing treatment in the oncology department or day care unit at the TASMC oncology division. From January 2015 to September 2016, upon arrival for treatment, patients were invited to voluntarily complete a hospital-approved anonymous questionnaire for evaluating their attitudes toward clinical trials, and the level of their understanding of relevant key concepts. Eligible patients were Hebrew-speaking, 18 years of age or older and willing to sign a written consent form. The questionnaire’s structure was based on others in the literature [14, 25, 26] and adapted for the Israeli population (Appendix A). It was comprised of three parts: the first evaluated demographic information and past experience in clinical research, and the second was composed of a 21-item true/false test on basic knowledge regarding clinical trials, including methods, goals and expectations. In the third section, the patients were asked whether they would agree or decline to participate in a clinical trial were they offered to do so. Entering free text about their main concerns about participating in a clinical trial was optional. The questionnaires' simplicity and clarity were evaluated by 15 members of the medical staff from the TASMC. Internal validation was performed by utilizing the α-Cronbach test on a pilot cohort of 50 oncologic patients, and further confirmed on a final cohort of 200 participants, with an excellent score set at 0.909 on the α-Cronbach test. A possible bias to the study was that the included population had to speak fluent Hebrew and did not include a fairly large portion of the population, such as former Soviet immigrants or non-native-speaking Israeli Arabs. In addition the stage of the disease and the prognosis may also have an effect..

### Statistical analysis

Patients’ characteristics were presented using descriptive statitsics: Mean and standard deviations for continuous variables and counts and percentages for categorical variables. A score of 60% correct answers on the knowledge test was considered a “high level of knowledge” and a score lower than 60% was considered “low level of knowledge”. Comparison of variables between two groups were tested with Fisher’s exact test for binary variables, with the non-parametric Mann-Whitney test for continuous variables. Statistical significance of all tests was defined at the α = 0.05 level, and all tests were 2-sided. Internal consistency was evaluated by the α-Cronbach measure. All analyses were performed using case-wise deletion, under the missing completely at random (MCAR) assumption.

## Results

A total of 200 patients completed the questionnaires out of 235 patients that were invited to participate, representing an 85% response rate. The main reason given for refusal was lack of time. The mean ± standard deviation age of the participants was 58.3±12.8 years, and 45.9% (n = 90) were males. The most common cancer sites were breast (21.9%), colorectal (17.2%) and lung (10.4%). Almost one-half of the population (49.2%, n = 93) was born in Israel, defined themselves as being non-religious (62.3%, n = 124) and had some level of academic education (43.7%, n = 86). The questionnaire included the official Israeli government-issued statistic on average monthly income in New Israeli Shekels and 100 participants (50%) reported that their income level ranged from average to high. Most participants reported living with a partner (70.9%, n = 141, Table 1).

**Table 1:** Participant characteristics according to past experience in clinical trials

Characteristic		All participants		Past clinical research experience		
				No/not sure, n	Yes, n	p value^*^
Total		200	100%	151 (76.6%)	46 (23.4%)	
Mean age (years)		58.3 ± 12.8				
Sex	Male	90	45.90%	65 (43.3%)	24 (54.5%)	0.189
	Female	106	54.10%	85 (56.6%)	20 (45.4%)	
Birthplace	Israel	93	49.2%	61 (41.5%)	31 (68.9%)	0.744
	Russia^†^	18	9.5%	86 (58.5%)	14 (31.1%)	
	Europe	56	29.6%			
	Africa	11	5.80%			
	Asia	5	2.6%			
	America	6	3.1%			
Education	Elementary/Junior high,	15	7.60%	94 (63.1%)	15 (32.6%)	<0.0001
	Professional school /agricultural	23	11.70%			
	High school	22	11.20%			
	Yeshiva (religious college)	5	2.50%			
	Non-academic training	46	23.40%			
	Academic	86	43.70%	55 (36.9%)	31 (67.4%)	
Religion	Secular (non-religious Jewish)	124	62.30%	88 (58.3%)	35 (76.1%)	0.037
	Observant	50	25.10%	63 (41.7%)	11 (23.9%)	
	Religious	15	7.50%			
	Orthodox	4	2.00%			
	Other	6	3.00%			
Residential Status	Alone	35	17.60%	22 (14.6%)	13 (28.3%)	0.046
	With family/partner	141	70.90%	129 (85.4%)	33 (71.7%)	
	With caregiver	10	5.00%			
	Other (nursing home, etc.)	13	6.50%			
Income	Much more than average	8	4.10%	36 (24.2%)	19 (42.2%)	0.194
	More than average	47	24.00%			
	Average	45	23.00%	80 (53.7%)	17 (37.8%)	
	Less than average	31	15.80%			
	Much less than average	23	11.70%			
Perception of understanding how clinical trials work	Agree/strongly agree	92	47.70%	54 (36.5%)	38 (84.4%)	<0.0001
	Neutral/disagree/strongly disagree	101	52.30%	94 (63.5%)	7 (15.6%)	
Agree to participate	Yes	120	63.80%	66 (44.9%)	33 (73.3%)	0.01
	No	67	35.60%	81 (55.1%)	12 (26.7%)	
Cancer site	Breast	42	21.90%			
	Colorectal	33	17.20%			
	Lung	20	10.40%			
	Prostate	10	5.20%			
	Other	87	45.30%			

^*^Compared between the groups with and without experience in clinical trials.

^†^Provided separately because of the large proportion of Russian-born participants

Nearly one-half of the surveyed population (47.7%, n = 92) agreed or very much agreed that they have a high level of understanding of how clinical trials work. The majority of the study population (63.8%, n = 120) would agree to participate in a clinical trial were they offered to, while 35.6% (n = 67) would decline.

### Analysis of past experience in clinical trials

Forty-six (23.4%) of the respondents had participated in a clinical trial in the past. Most of them had an academic education compared with those who had no experience in clinical trials (67.4%, n = 31 vs. 36.9%, n = 55, respectively, p < 0.0001), the majority of them defined themselves as secular (non-religious Jews; 76.1%, n = 35 vs. 58.3% religious Jews, n = 88; p = 0.037) and were more likely to live alone rather than with a partner or caregiver (28.3%, n = 13 vs. 14.6%, n = 22, respectively, p = 0.046). As expected, patients with clinical trial experience were significantly more likely to define themselves as having a good understanding of how medical research works, compared to those without experience (84.4%, n = 38 vs. 36.5%, n = 54, p < 0.0001). They were also more inclined to participate in a future study, compared to those with no experience (73%, n = 33 vs. 44.9%, n = 66, p = 0.01). The participants’ demographic characteristics and analysis of their responses according to past experience in clinical trials are presented in Table 1.

### Analysis according to level of knowledge

The vast majority of the participants (164 out of 200, 82%) did not meet the minimum knowledge level on the knowledge test, with similar proportions of high- vs. low-level knowledge among Israeli-born and non-Israeli-born respondents. There were notable differences between the high- and low-level knowledge groups. Those with high-level knowledge were more likely to have an academic education compared with those with low-level knowledge (66.7%, n =24 vs. 38.5%, n = 62, respectively, p = 0.003). The proportion of patients with a "much higher than average" level of income was higher for the high–level knowledge group (11.5%, n = 4 vs. 2.5%, n = 2, p = 0.039).

**Fig. 1:**
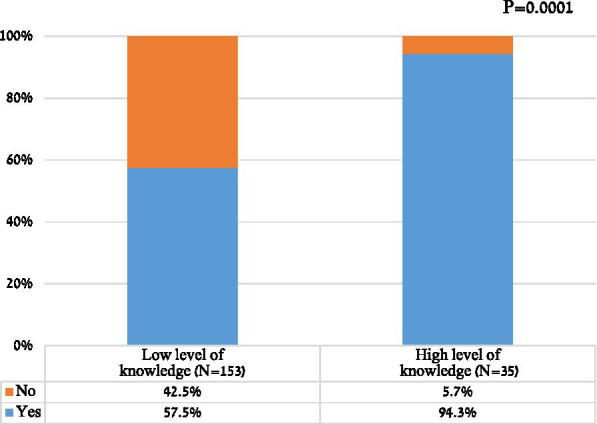
Willingness to participate in future clinical research according to knowledge test score; a score of 60% correct answers were considered as a passing mark.

As expected, the high-level knowledge group contained a significantly larger proportion of patients with past experience in clinical trials compared with the low-level knowledge group (47.2%, n = 17 vs. 18%, n = 29, p < 0.0001). Also as expected, those with high-level knowledge were significantly more interested in participating in a future clinical trial than those with low-level knowledge (94.3%, n = 33 vs 57.5%, n = 88, p < 0.0001; Fig. 1). Interestingly, patients with high-level knowledge who had no past experience in clinical research were more inclined to participate in a clinical trial compared to those with low-level knowledge (94.4%, n = 17 vs 50.8% n = 64, p < 0.0001) (Table 2).

**Table 2:** Expressed willingness to participate in clinical trials according to past experience in a trial and score on the knowledge test (n = 186)

Past Score on knowledge test experience		Willingness to participate		
		Yes	No	p value
No past research	Low-level knowledge	64 (50.8%)	62 (49.2%)	p<0.0001
	High-level knowledge	**17 (94.4%)**	1 (5.6%)	
Past research	Low-level knowledge	24 (96%)	1 (4%)	p=0.055
	High-level knowledge	16 (94.1%)	1 (5.9%)	

**Bold** indicates significance.

The patients who would decline to participate in clinical trials were asked to rate the importance of selected parameters that would influence their decision. Although the difference between the two knowledge groups regarding the fear of receiving a placebo drug and not the experimental drug did not reached a level of significance a trend were seen; the majority of both high and low-level knowledge patients considered the argument as "not important" or as "important", respectively (p = 0.055 Mann-Whitney U Test).

### Major misconceptions and common knowledge

We analyzed our questions according to the rate of correct answers for each question. Questions that had 80% correct answers represented “common knowledge”, and questions that had a maximum of 40% correct answers represented the most frequent misconceptions. According to our questionnaire, common knowledge in the study’s population consisted of: 1. The aims of a clinical trial were to evaluate the safety and the efficacy of an experimental drug, and 2. A new drug must be studied in a preclinical setting prior to the administration to a human patient. Common misconceptions that were revealed from this analysis were: 1. Receiving a placebo precludes receiving the standard of care; 2. Signing an informed consent abolishes the possibility of a future refusal; 3. The choice of the experimental drug depends entirely upon the oncologist’s preferences; 4. The recruiting physician personally benefits from the patient’s enrollment into clinical trials.

## Discussion

The results of this study outline the attitude towards clinical trial participation of oncologic patients from a large tertiary referral hospital in Israel with an active clinical trial unit. 35.6% of this study population predicted that they would decline to be enrolled in a clinical trial should they be offered an opportunity to participate. Our results are in line with those of Klabunde et al. who evaluated factors influencing accrual in a large National Cancer Institute cohort and found that approximately 40% of the clinically eligible patients refused to be enrolled into a study [27]. Although many respondents in several large trials made by the CISCRP perceived clinical trials as highly important, their actual understating of the trials was very limited.

In accordance with other studies [9–11], we found a strong correlation between patients' willingness to participate in a study and specific socioeconomic characteristics: the typical consenting patient was more likely to have an academic level of education and to have an average or above-average income. Neither sex nor place of birth had any significant effect. Interestingly, we found that patients who live alone were more likely to consent to participate. In addition, the level of religious observance also seemed to be related with attitude towards participation in clinical trials: secular Jews were more open to doing so than orthodox Jews. This may be related to possible reluctance to take risks that could potentially shorten life [28].

Importantly, we found a discrepancy between the self-reported level of familiarity with how clinical research works and the actual knowledge as demonstrated in the knowledge test. More than one-half of the population in the study was convinced that they had a high level of understanding, yet the majority of them performed poorly on the knowledge test. Other studies showed similar results when using different questionnaires and scoring systems [14, 29, 30]. As hypothesized, we found that patients with higher levels of knowledge were more likely to be willing to participate in clinical trials, and the difference was statistically significant (Fisher exact test, p < 0.0001).

Wide information gaps and misconceptions that were crucial for the decision-making process were identified among the patients who had poor results on the knowledge test. Specifically, the concepts of “placebo” and “standard of care” were poorly understood, and the informed consent procedure was perceived as being obligatory and non-rescindable. An especially worrisome result was the erroneous belief that the treating physician personally benefits from patient enrollment. Such disturbing perceptions of the levels of ethical adherence on the part of physicians might partially explain the hesitant attitudes that patients demonstrate toward clinical trials.

When considering participation in a clinical trial, patients have to face more than a few uncertainties, most of which are based on their knowledge and perceptions that ultimately dictate their decision. The findings of our study are in line with earlier reports on the advantages of providing accessible clinical trial-related information to potential participants [21–24]. The findings of the current survey suggest that such interventions should focus on developing patient education strategies that could minimize specific gaps in knowledge. They support previous observations on the important role of the physician in communicating the correct essential information on clinical trials [21–24]. Following our observations in this study we plan to suggest several changes in our daily work regarding clinical trials. first and for all we wish to educate both the oncologists and the patients in our oncology division about the essence of clinical studies. We believe s that by doing so we have the potential to make some changes in our study practice and increase the recruitment rate. We may also give more patients a chance to be exposed to novel therapeutic drugs and procedures as well as additional hope.

### Strengths and limitations of this study

Our study adds to the accumulating evidence of patient participation in oncologic clinical trials, and provides data to explain why so many patients are reluctant to do so. Our study was limited by the fact that participants were recruited in a single center who may not adequately represent the entire patient population nationwide. Furthermore, our study participants were Hebrew-speaking, whereupon certain subgroups of the Israeli society may not have been well-represented.

### Conclusion

In conclusion, the findings of this study suggest that patient reluctance to participate in clinical studies can be attributed in part to lack of knowledge regarding the clinical research system and, specifically, the rights of study participant and the ethical obligations of the physician. This insight can form the basis of a paradigm of patient as well as physician education, starting from an early course of the disease that may enhance the rates of enrolment into oncologic clinical trials.

### Practice implications

Our results suggest that the best practice for recruiting oncologic clinical trial participants involves educating patients about clinical research, explaining the lack of vested interest on the part of the treating oncologist, assuring the patient's ability to withdraw consent at any time and for any reason and explaining the implications of assignment to the control arm of a study.

## Data Availability

The datasets generated and/or analysed during the current study are not publicly available due to patient's privacy. Personal patient information was anonymized and stored under a password-protected computer. The computer is located at a locked office of the investigator. The data that support the findings of this study are available from Dr. Ravit Geva but restrictions apply to the availability of these data, which were used under license for the current study, and so are not publicly available. Data are however available from the authors upon reasonable request and with permission of Tel Aviv Sourasky Medical Center Helsinki Committee.

## Appendix A – Questionnaire on Knowledge about Medical Research (adapted to the Israeli setting)

Date:

You are requested to fill out the following anonymous questionnaire on "Oncology Patient Participation in Clinical Studies." We request your consent that the information you provide will be used for research to be conducted within our oncology department as approved by the institutional ethics committee and hospital management. You can choose whether or not you wish to respond to items in the questionnaire. Your cooperation is highly appreciated.

## Abbreviations

CISCRP: Center for Information and Study on Clinical Research Participation
TASMC: Tel Aviv Sourasky Medical Center
